# A 10-year update to the principles for clinical trial data sharing by pharmaceutical companies: perspectives based on a decade of literature and policies

**DOI:** 10.1186/s12916-023-03113-0

**Published:** 2023-10-23

**Authors:** Natansh D. Modi, Ganessan Kichenadasse, Tammy C. Hoffmann, Mark Haseloff, Jessica M. Logan, Areti A. Veroniki, Rebecca L. Venchiarutti, Amelia K. Smit, Haitham Tuffaha, Harindra Jayasekara, Arkady Manning-Bennet, Erin Morton, Ross A. McKinnon, Andrew Rowland, Michael J. Sorich, Ashley M. Hopkins

**Affiliations:** 1 College of Medicine and Public Health, Flinders University, Adelaide, SA Australia; 2 Flinders Centre for Innovation in Cancer, Department of Medical Oncology, Flinders Medical Centre, Adelaide, SA Australia; 3 Institute for Evidence-Based Healthcare, Faculty of Health Sciences and Medicine, Bond University, Gold Coast, QLD Australia; 4 Adelaide, Australia; 5 Clinical and Health Sciences, University of South Australia, Adelaide, SA Australia; 6 Institute of Health Policy, Management and Evaluation, University of Toronto, Toronto, Canada; 7 Knowledge Translation Program, Li Ka Shing Knowledge Institute, St. Michael’s Hospital, Toronto, Canada; 8 Sydney School of Public Health, Faculty of Medicine and Health, The University of Sydney, Camperdown, NSW Australia; 9 Department of Head and Neck Surgery, Chris O’Brien Lifehouse, Sydney, NSW Australia; 10 The Daffodil Centre, The University of Sydney, A Joint Venture with Cancer Council NSW, Sydney, NSW Australia; 11 Centre for the Business and Economics of Health, The University of Queensland, Brisbane, QLD Australia; 12 Cancer Epidemiology Division, Cancer Council Victoria, Melbourne, VIC Australia; 13 Centre for Epidemiology and Biostatistics, Melbourne School of Population and Global Health, The University of Melbourne, Melbourne, VIC Australia; 14 School of Public Health and Preventive Medicine, Faculty of Medicine, Nursing and Health Sciences, Monash University, Clayton, VIC Australia

**Keywords:** Data sharing, Pharmaceutical industry, PhRMA/EFPIA, Transparency

## Abstract

Data sharing is essential for promoting scientific discoveries and informed decision-making in clinical practice. In 2013, PhRMA/EFPIA recognised the importance of data sharing and supported initiatives to enhance clinical trial data transparency and promote scientific advancements. However, despite these commitments, recent investigations indicate significant scope for improvements in data sharing by the pharmaceutical industry. Drawing on a decade of literature and policy developments, this article presents perspectives from a multidisciplinary team of researchers, clinicians, and consumers. The focus is on policy and process updates to the PhRMA/EFPIA 2013 data sharing commitments, aiming to enhance the sharing and accessibility of participant-level data, clinical study reports, protocols, statistical analysis plans, lay summaries, and result publications from pharmaceutical industry-sponsored trials. The proposed updates provide clear recommendations regarding which data should be shared, when it should be shared, and under what conditions. The suggested improvements aim to develop a data sharing ecosystem that supports science and patient-centred care. Good data sharing principles require resources, time, and commitment. Notwithstanding these challenges, enhancing data sharing is necessary for efficient resource utilization, increased scientific collaboration, and better decision-making for patients and healthcare professionals.

## Background

Clinical trial data sharing is vital for fostering transparency, quality, scientific advancement, reducing research waste, and sustaining confidence in the pharmaceutical industry. In 2013 [1], a large proportion of the industry, through the Pharmaceutical Research and Manufacturers of America (PhRMA) and European Federation of Pharmaceutical Industries and Associations (EFPIA), endorsed a commitment to:

1. Share participant-level data, study-level data, and protocols from clinical trials of United States (US) and European Union (EU) registered medicines with qualified researchers
2. Provide public access to clinical study reports (CSR), at minimum synopses, from clinical trials submitted to the Food and Drug Administration (FDA), European Medicines Agency (EMA), and EU Member States
3. Share summary result reports with clinical trial participants
4. Establish public web pages displaying the companies’ data sharing policies and procedures
5. At a minimum, publish results from all phase 3 and any clinical trial of significant medical importance

PhRMA and EFPIA members are currently at the forefront of data sharing commitments, surpassing academia, and statutory requirements. However, there is still room for further improvement and standardization of commitments to enhance communication of clinical trial results with the public, as well as to facilitate a more efficient data sharing ecosystem.

## Progress and challenges in clinical trial data sharing

**Fig. 1:**
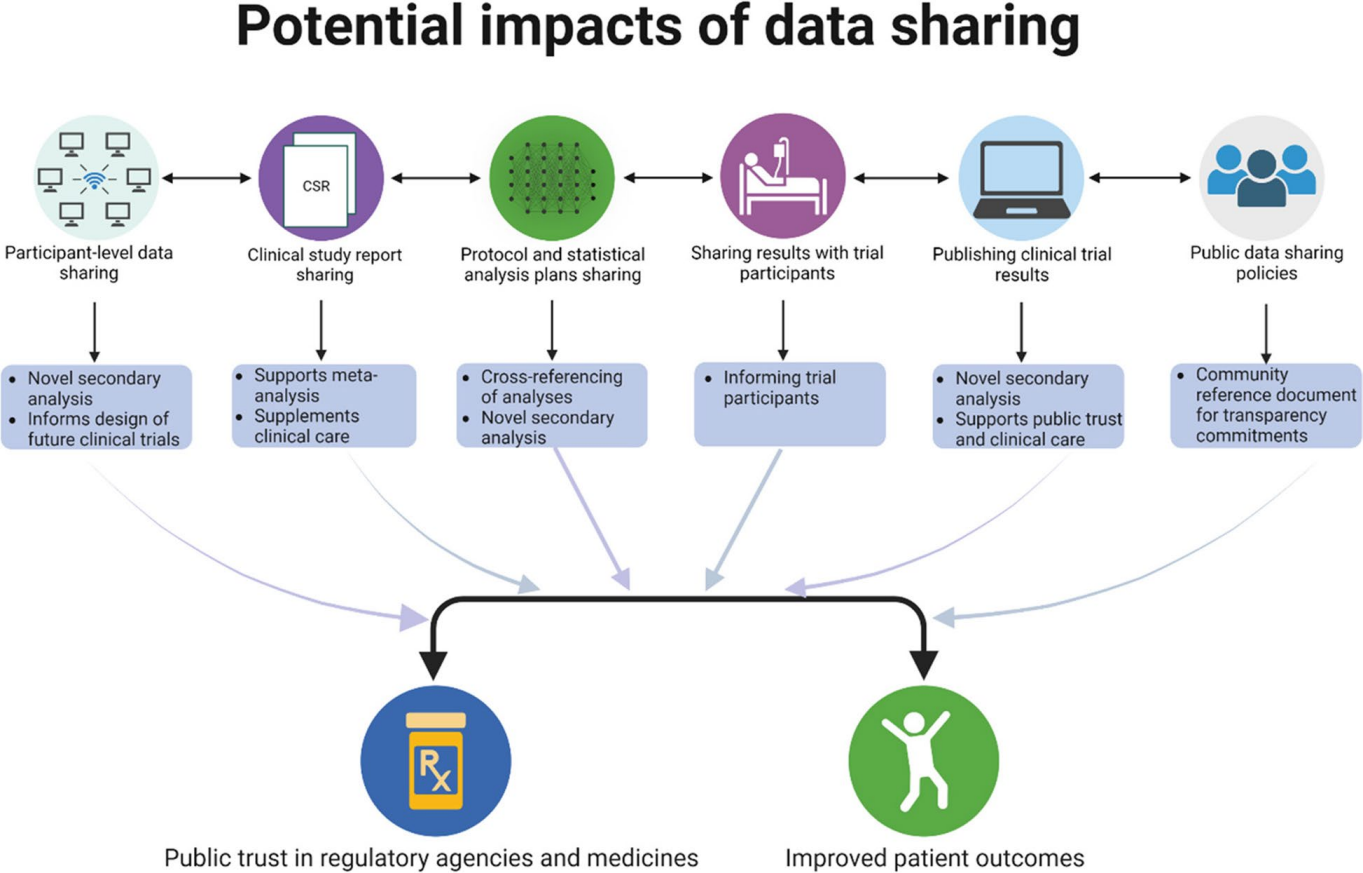
Potential impacts of data sharing

The PhRMA/EFPIA commitments marked significant progress in providing clinical trial results to participants and the general public, as well as in establishing a data sharing ecosystem that enriches the post-approval evidence base through open research conducted by independent researchers (Fig. 1) [2–7]. With 18 of the current top 20 pharmaceutical companies by revenue being PhRMA/EFPIA members, the commitment holds significant weight [8]. Moreover, 15 of the top 20 companies are also TransCelerate (a collaborative network of pharmaceutical companies) members, ensuring access to guidance on collecting trial data under standardised quality conditions from the outset [9]. However, recent investigations indicate that over 50% of the clinical trials supporting the FDA approval of 115 anticancer medicines over the past 10 years were ineligible for participant-level data sharing [8]. This finding includes 90% of the clinical trials summarised in the product labels of nivolumab, pembrolizumab, and pomalidomide—this is concerning as these medicines currently rank in the top 10 anticancer medicines by global sales. Furthermore, investigations indicate that much of the participant-level data underpinning the FDA/EMA approval of COVID-19 vaccines is currently out of scope for request and will likely remain so for some time [3]. The above findings underscore an urgent need for improvements in participant-level data transparency, especially for pivotal medicines with significant medical importance.

Since 2013, policies and recommendations for sharing specific data elements have been developed by various organizations, including the FDA, EMA, Health Canada, World Health Organization (WHO), US National Institutes of Health (NIH), Institute of Medicine (now the National Academy of Medicine), White House Office of Science and Technology Policy, International Committee of Medical Journal Editors (ICMJE), Bill and Melinda Gates Foundation, Wellcome Trust, and the GO FAIR Initiative, among others, highlighting significant developments in the data sharing landscape [10–23]. Despite these developments, the 2013 PhRMA/EFPIA principles still serve as a significant point of reference within the data sharing policy web pages of many pharmaceutical companies [1].

## Enhancing data sharing practices

Drawing on a decade of literature and policy developments, this article presents perspectives from a multidisciplinary team of authors, including researchers, clinicians, and consumers. The article works towards proposing evidence-based recommendations for potential updates to the pharmaceutical industry data sharing principles established in 2013. Our primary aim was to review the current literature to identify and highlight feasible, urgent next steps for enhancing the data sharing ecosystem and for promoting harmonised data sharing practices among companies. We have formulated these recommendations based on current literature and reported experiences. However, we acknowledge that they may not address all the challenges at hand, and continued progress will still be necessary.

Table 1 presents our recommended updates, which aim to enhance existing principles, promote harmonised data sharing practices, and establish clearer guidelines regarding which data should be shared, when it should be shared, and under what conditions. The goal is to foster the data sharing ecosystem [24, 25]. Exemplifying the feasibility of the recommendations presented in Table 1, most are currently implemented in a fragmented manner across companies. While the primary focus of this manuscript is on pharmaceutical industry data sharing practices, the perspectives are also relevant to non-industry trial sponsors and investigator-initiated trials. Additionally, this manuscript is expected to be particularly valuable for smaller pharmaceutical companies that have less established data sharing practices [24]. Below, we outline the key literature and policy developments justifying our recommendations.

**Table 1:** Recommendations for updating data sharing policies to address emerging policy and research developments from the past decade

Summary of 2013 PhRMA/EFPIA principles	Recommended updates to the principles
**Participant-level data sharing with researchers**	
• Pharmaceutical companies commit to sharing with qualified researchers’ patient-level data from clinical trials for medicines and indications approved in the US and EU. • Each company will establish a scientific review board who are not employees of the company. • Data requests will be evaluated against a description of the data being requested, hypothesis being tested, research rationale, analysis plan, publication and posting plan, qualifications and experience of the team, and a description of conflicts of interest, including potential competitive use of the data and the source of any research funding. • Companies will implement a system to provide applicable data and protocols to help facilitate the research.	1. Participant-level data from any clinical trial result submitted to support drug approvals should be eligible for sharing (irrespective of continuing follow-up). Companies should endeavour to facilitate the sharing of clinical trials not directly supporting medicine approvals within a clearly defined timeframe of primary result completion/publication. 2. Companies should aim to only assess if trials are in scope for participant-level data sharing. All decisions on the legitimacy of a data request should be evaluated by an independent scientific review panel. 3. Companies should outline the date on which their trial consent procedures were last updated and provide an example form to avoid issues with future data sharing. 4. Companies should maintain public lists of sponsored trials that are eligible/ineligible for participant-level data sharing. 5. Where possible, companies should provide full CSRs, data dictionaries, data derivation documents, protocols, SAPs, and anonymisation guides with requests to help facilitate valid secondary research.
**Sharing of clinical study reports**	
• To help patients and healthcare professionals understand the results of clinical trials and the evidence used to approve a new medicine (US and EU), pharmaceutical companies will make publicly available, at a minimum, the synopses of CSRs for clinical trials. • Companies will evaluate requests for full CSRs.	1. While initiatives to share result synopses are admirable, given the extent of extra clinical information and detail contained in CSRs, full CSRs from all clinical trials submitted to support medicine approvals should be publicly available for download. 2. Subsequent versions of CSRs should be made available when prepared. 3. Both the FDA and EMA have acknowledged resource difficulties in disseminating CSRs; thus, it is likely companies need to engage in processes that facilitate public downloads.
**Sharing of protocols and statistical analysis plans**	
• Pharmaceutical companies commit to sharing with qualified researchers’ protocols from clinical trials for medicines and indications approved in the US and EU.	1. Companies need to make SAPs and protocols of all published clinical trials publicly available, and consideration should be given to sharing within 6 months of enrolling the first participant. 2. Updated versions of SAPs and protocols should be available when prepared.
**Sharing results with trial participants**	
• To help inform and educate patients about the clinical trials in which they participate, pharmaceutical companies will work with regulators to adopt mechanisms for providing a factual summary of clinical trial results to research participants.	1. All trial participants should be provided a lay summary reporting trial results within 12 months of primary outcome completion. These lay summaries should also be made publicly available at that time. 2. Subsequent summaries should be prepared for follow-up outcomes. 3. Study protocols should include plans for lay summaries.
**Publishing clinical trial results**	
• All clinical trials should be considered for publication irrespective of whether the results were positive or negative. At a minimum, results from all phase 3 trials and any trial results of significant importance should be published.	1. All clinical trials must have result summaries published to the trials registry site within 12 months of the primary outcome completion, with efforts to make a scientific journal publication available within the same timeframe. 2. Result summaries and scientific journal publications should occur for follow-up outcomes. 3. Publishing of clinical trial results should occur regardless of study outcomes or phase. 4. Study protocols should include plans for publications.
**Public data sharing policies**	
• Companies following the 2013 PhRMA/EFPIA Principles for Responsible Clinical Trial Data Sharing will certify on a publicly available website that they have established policies and procedures to implement these data sharing commitments.	1. Towards harmonising terminologies and processes, companies should have public data sharing policies providing precise and detailed information on policies and procedures (including web links for access) to sharing participant-level data, full CSRs, protocol/SAPs, lay summaries, CSR synopses, reporting of results on clinical trial registries, and scientific journal publications. 2. Policies should be written with subheadings and numbered criteria, providing clear information on what data will be shared, when, and under what conditions for each data item. 3. To facilitate cross-referencing between documents, clinical trial registration and internal trial numbers/names should be included in all publications, product information leaflets, participant-level data, CSRs, protocols/SAPs, and lay summaries.

*CSRs* Clinical study reports, *EFPIA* European Federation of Pharmaceutical Industries and Associations, *EMA* European Medicines Agency, *EU* European Union, *FDA* Food and Drug Administration, *PhRMA* Pharmaceutical Research and Manufacturers of America, *US* United States

## Participant-level data sharing

Transparent sharing of participant-level data facilitates novel secondary analyses, avoids unnecessary study duplication, and informs future trial design [2–7]. Participant-level data from clinical trials of newer medicines are vital as they are the centrepiece of safety and efficacy for these medicines [3, 26]. The EMA has indicated that they will implement future policies to promote participant-level data sharing [27], albeit, no US or EU regulations currently mandate participant-level data sharing from industry-sponsored medicine trials.

Nonetheless, most large pharmaceutical companies have processes to share participant-level data [8]. However, recent research indicates that approximately 50% of participant-level data supporting newly registered medicines are not eligible (i.e., in scope) for request [3, 8, 28, 29]. Specific trials are often deemed ineligible for sharing due to ongoing follow-up, extended embargos, requirements for both EMA and FDA approval, and issues related to the need for explicit informed consent from study participants [3, 8, 28, 29]. To expand data sharing, research suggests that participant-level data from any clinical trial underpinning a product label or submitted to the FDA or EMA for drug approval should be immediately eligible for sharing [3, 8]. Sharing this participant-level data should not be restricted by the clinical trial having long-term follow-up. While long-term follow-up is crucial to understanding longer-term safety and efficacy, it should not prevent the sharing of result data that are responsible for the medicines approval [3, 8]. Pharmaceutical companies should also facilitate the sharing of clinical trials that do not directly support medicine approvals, within a well-defined timeframe after the primary results are completed or published to reduce research waste [3, 8].

Decisions on the legitimacy of independent data requests, including the hypotheses tested, the research rationale, the analysis plan, the publication plan, and the qualifications of the research team, should be made by independent scientific review panels [21]. To facilitate these review processes, it is important to establish mechanisms that provide training to independent individuals, enabling them to develop a deep understanding of the technical, legal, and scientific aspects required to assess data requests [30, 31]. The objective is to establish a pool of independent reviewers, enabling pharmaceutical companies to limit their role to simply determining the sharing eligibility of the requested participant-level data. Towards this, pharmaceutical companies should be aiming to maintain up-to-date, publicly accessible registers documenting the sharing eligibility of their clinical trials [10]. This should include a specific indication of clinical trials that are ineligible, along with clear reasons outlining why and when trials will become eligible. Among the various reasons for ineligibility, consent form issues have been identified as a major concern. To this issue, company web pages should provide clear information on updated consenting procedures, along with consent form examples [10].

Data protection and security must be a top priority for all parties, including the requestor [32]. Participant-level data sharing typically takes place on platforms requiring rigorous assessment of the requesting teams’ qualifications [8, 21]. Researchers often obtain access to data in a secure, password-protected research environment from which data cannot be downloaded locally [21]. The procedures for anonymising data should align with the level of protection required. Procedures that redact key information (such as survival and adverse event data) for secondary research should be evaluated for appropriateness and necessity [21, 32]. Furthermore, to facilitate the valid use of participant-level data, companies should enhance the findability and accessibility of clinical study reports, annotated case report forms, data dictionaries, data derivation documents, protocols, statistical analysis plans, and anonymisation guides. Such transparency, as highlighted by the FAIR (Findable, Accessible, Interoperable, and Reusable) data principles, is essential for enabling independent researchers to create detailed data requests and verify their data preparation processes when undertaking participant-level data analyses [10, 33, 34].

Independent researchers should also be committed to publishing their analyses, sharing code for reproducibility, maintaining data confidentiality, not disclosing data to unauthorised parties, and not attempting to re-identify study participants [1, 35]. Acknowledgements to data contributors and original investigators should be made in all secondary data use publications, and researchers should recognise that original investigator contributions may warrant authorship on new work [36].

## Sharing of clinical study reports

CSRs are standardised documents that contain detailed information (often > 1000 pages) on study designs and study-level results from clinical trials, providing vastly more detail than either clinical trial result synopses or publications [37–40]. Given their comprehensive and high-quality nature, CSRs are a valuable resource for research, especially for meta- and patient-level data analyses. Furthermore, they can aid healthcare providers in making informed decisions for at-risk individuals—which can be particularly important for understanding toxicity likelihoods with newer medicines [38, 41, 42].

CSRs are often prepared as supporting documents for medicine submissions to approval and reimbursement bodies. CSR transparency has been acknowledged by the EMA, Health Canada, and the FDA as a mechanism to support public trust in regulatory processes [17, 18, 41, 43]. Both the EMA and Health Canada have regulations stating that they will publicly share CSRs submitted to them that support medicine approval decisions [18, 43]. However, resource difficulties have hindered the EMA in disseminating CSRs, and they have not been doing so since 2018 [17, 43]. Meanwhile, the FDA has no CSR sharing policy and instead encourages sponsors to voluntarily disclose such information due to the logistic challenges it would face in implementing such a process [17, 43].

While initiatives to publicly share result synopses and publications are commendable, our evaluations suggest that full CSRs from all clinical trials submitted to support medicine approvals should be publicly available for direct download, irrespective of whether the trial has continuing follow-up. Additionally, subsequent versions of CSRs should be made available as they are prepared, as new reports are often created for later data cuts. Given that there are functionalities to upload supporting documents (such as CSRs) on clinical trial registration websites [15, 44], this could be a future option for voluntary disclosure. Furthermore, while ensuring patient anonymity is critical, companies should not endorse the practice of over-redaction in their CSR anonymisation processes [45–47].

## Sharing of protocols and statistical analysis plans

Statistical analysis plans (SAPs) and protocols are essential resources for cross-referencing planned analyses and reporting of outcome/adverse event measures from clinical trials [48]. They also provide researchers with a thorough understanding of the data gathered during a clinical trial, facilitating the design of secondary data analyses [19].

The ICMJE recommends that SAPs and protocols should be reviewed when evaluating journal submissions and be made publicly available upon publication [20]. Similarly, NIH regulations (effective from 2017) indicate that SAPs and protocols should be publicly available at the time of publishing summary results [11, 14]. Notably, in 2020, both Moderna and Pfizer released detailed protocols for their COVID-19 vaccine trials, well before publishing the results [49]. We propose that companies should publicly share SAPs and protocols for all published clinical trials and consider sharing them within 6 months of enrolling the first participant. Functionalities to upload SAP and protocol documents are available on clinical trial registries [15, 44]. Subsequent versions of SAPs and protocols should be made available when prepared (i.e., updates occur). Data management and data sharing plans should be outlined in SAPs and protocols [50].

For secondary analyses of shared data, academic institutions and data sharing platforms should have public processes for documenting approved SAPs and requests.

## Sharing results with trial participants

Lay summary documents (or plain language summaries) are reports that convey clinical trial results in a simplified format for study participants and the general public [51, 52]. Sharing of such documents is recognised by regulators and companies as a mechanism to enhance public trust in medicines [52–54]. The Declaration of Helsinki (2013) mandates that all participants ‘should be given the option of being informed about the general outcome and results of the study [55].’

Companies should meet the lay summary requirements of the European Union Clinical Trials Regulation (EU CTR) 536/2014 (effective January 2022) [16, 54]. The regulation states, and we support, that all clinical trial participants should be provided a lay summary reporting the results of the clinical trial within 12 months of primary outcome completion [16, 54]. Subsequent summaries should be prepared for collected follow-up data. EU CTR indicates all lay summaries should be made publicly available. Towards best practices, preparation, and dissemination plans for lay summaries should be included in study protocols [52].

## Publishing clinical trial results

The Declaration of Helsinki (2013) mandates that results from human studies should be made publicly available [55]. US and EU regulations now require the publishing of clinical trial result summaries to ClinicalTrials.gov and the Clinical Trial Information System, respectively, within 12 months of primary outcome completion [13, 14, 56]. Requests have also been made to make scientific journal publications available in the same timeframe [22]. We propose that the dissemination of result publications should not depend on clinical trial outcome or phase [22] and should cover all follow-up data. Furthermore, consistency of results presentations between publications, regulatory evaluations, and product information leaflets should be ensured [57].

## Public data sharing policies

Pharmaceutical companies should have publicly available web pages detailing their data sharing policies, procedures, and commitments [1]. Detailed public policy information has been linked to improved clinical trial transparency [8, 24, 28, 58]. Table 1 outlines our perspectives on essential policy updates for data sharing based on emerging literature over the past decade. To implement these updates, companies should establish clear public policies for sharing participant-level data, full CSRs, protocol/SAPs, lay summaries, CSR synopses, reporting of results on clinical trial registries, and journal publications [19]. These are among the critical domains of data sharing advocated by the (now) National Academy of Medicine [19].

We recommend that data sharing policies should be written in a standardised format, including sub-headings for each data item, with numbered criteria for easy referencing by independent scientific review panels. Public registers of data sharing requests and decisions should be kept up-to-date [21]. Additionally, companies should have a register of their clinical trials that are eligible for data sharing and those that are not [10]. The register should specify the eligibility criteria and procedures for accessing participant-level data, full CSR, protocol/SAPs, lay summary, CSR synopsis, reporting of results on clinical trial registries, and scientific journal publications for every clinical trial [10, 19].

To facilitate cross-referencing and linkage between documents, company processes should aim to include both clinical trial registration numbers and internal trial numbers/names in all publications, product information leaflets, participant-level data, CSRs, protocols/SAPs, and lay summaries [10, 59]. This cross-referencing between documents is currently undertaken poorly by most companies.

## Future directions

While the primary aim of the article was to highlight feasible, urgent next steps for enhancing the data sharing ecosystem, we acknowledge that continued progress will still be necessary even if all the recommendations put forward are adopted. Looking ahead, the clinical trial data sharing landscape holds tremendous potential for fostering new scientific discoveries and informing decision-making [60–63]. Notably, Vivli alone as a participant-level data sharing platform has facilitated the publication of over 180 research works over the past 5 years, an output that has increased from 2 manuscripts in 2019 to 85 in 2022 [64]. However, to fully realise the potential impact of the data sharing ecosystem, it will be important for all clinical trial sponsors and investigators, including non-industry trial sponsors, to take significant steps in improving standards.

It is acknowledged that at present the data sharing landscape is fragmented in many aspects [65]. In the future, there is hope for better utilization of public clinical trial registries as valuable resources for prospectively acknowledging the sharing eligibility of participant-level data, as well as facilitating public access to CSRs, protocol/SAPs, lay summaries, result publications, annotated case report forms, data dictionaries, data derivation documents, and anonymization guides [66–68]. At present the reporting and accessibility of these documents is somewhat disparate between companies, and the sharing eligibility of participant-level data for specific clinical trials is often not outlined prospectively.

Another consideration is the potential to centralise or transition participant-level data sharing to more open-access models. Undoubtedly, needing to access different platforms/servers (e.g. CSDR [69] and Vivli [70]) is a limiter to the effectiveness of undertaking participant-level data meta-analyses for investigations involving multiple companies. Considerations should be given to whether more open models, could facilitate crowd-sourced insights as well as minimising administrative burdens. Nonetheless, even with such a system, there is still a need for mechanisms that ensure the quality of outputs.

To enhance data sharing practices, there is a need for better methods to assess and distinguish between good and bad data sharers. A valuable step towards achieving this would be the implementation of improved meta-metrics on clinical trial data sharing. Currently, the best option for comparing the transparency practices of pharmaceutical companies is ‘The Good Pharma Scorecard’ [71]; however, it primarily ranks policies rather than comparing the outputs and performances of the companies. It is suggested that ‘The Good Pharma Scorecard’ could be significantly enhanced by incorporating insights into meta-metrics such as the total number of data requests received, the number of approved requests, and the number of citable public outputs facilitated for each company. This would offer a more comprehensive and transparent evaluation of data sharing efforts, enabling better recognition of companies with commendable metrics, and encouraging others to meet the standards of their competitors.

## Conclusions

Data sharing plays a vital role in fostering scientific progress and supporting well-informed decisions in clinical practice. Table 1 presents policy and process updates that are our perspectives—as based on the literature—to the next steps to enhance accessibility and transparency of participant-level data, CSRs, protocol/SAPs, lay summaries, and result publications from clinical trials. Implementing these principles will require resources, time, and commitment, and we acknowledge that new issues and areas for improvement may arise [72–74]. Nonetheless, these achievable suggestions aim to facilitate the development of a data sharing ecosystem that prioritises science and patient-centred care. Meeting these commitments is in the best interest of all institutions involved in clinical trials, including companies, universities, PhRMA/EFPIA, medical societies, advocacy groups, regulators, funders, and journals, because the ultimate goal is to ensure efficient resource utilization, foster scientific advancement, and facilitate the best decisions for patients.

## Data Availability

Not applicable.

## Abbreviations

CSR: Clinical study report
EFPIA: European Federation of Pharmaceutical Industries and Associations
EMA: European Medicines Agency
EU CTR: European Union Clinical Trials Regulation
EU: European Union
FDA: Food and Drug Administration
ICMJE: International Committee of Medical Journal Editors
NIH: National Institutes of Health
PhRMA: Pharmaceutical Research and Manufacturers of America
SAP: Statistical analysis plan
US: United States
WHO: World Health Organization
